# Vitamin D Fortification Strategies and Policy Landscape in Selected European Countries

**DOI:** 10.3390/nu18081194

**Published:** 2026-04-10

**Authors:** Bartłomiej Czyżniewski, Jolanta Chmielowiec, Krzysztof Chmielowiec, Magdalena Gibas-Dorna

**Affiliations:** Institute of Health Sciences, University of Zielona Góra, Zyty Str. 28, 65-046 Zielona Góra, Poland; b.czyzniewski@inz.uz.zgora.pl (B.C.); j.chmielowiec@inz.uz.zgora.pl (J.C.); chmiele@vp.pl (K.C.)

**Keywords:** vitamin D, food fortification policy, European countries, fortification strategies

## Abstract

Background: Vitamin D deficiency remains a widespread public health issue in Europe, despite the availability of sunlight, dietary sources, supplements, and food fortification. National fortification strategies differ substantially in their regulatory approaches, food vehicles, and fortification levels, influencing the population’s vitamin D intake and status. Objective: The primary objective of this study was to map vitamin D food fortification policies across European Union (EU) Member States, European Free Trade Association (EFTA) countries, and the United Kingdom (UK), focusing on regulatory frameworks, eligible food categories, and implementation models. Methods: A structured review of national legislation and official guidance on vitamin D food fortification was conducted between December 2025 and March 2026 across EU Member States (*n* = 27), EFTA countries (*n* = 4), and the UK. For EU Member States, the framework established by Regulation (EC) No 1925/2006 was examined alongside national implementation measures. For EFTA countries and the UK, corresponding national legislation and official regulatory guidance were reviewed. Data were extracted on fortification policy status, eligible food categories, legal basis, and fortification levels. Targeted searches of PubMed and Scopus were performed to identify modeling studies and policy analyses supporting the interpretation of the findings. Results: Fortification policies show marked heterogeneity. Mandatory fortification is limited to a few countries and specific foods: Finland (homogenized skim milk), Sweden (low-fat milk, fermented dairy, plant-based alternatives, and fat spreads), Belgium (margarine and selected fats), and Poland (margarine and fat spreads). In most other European countries, vitamin D fortification is voluntary under EU legislation or equivalent national legislation, depending on market uptake. Food vehicles vary regionally, with Northern Europe extending fortification beyond fats to include fluid milk and plant-based drinks, whereas other regions mainly fortify margarines, cereals, dairy products, and plant-based beverages. Fortification levels also differ, with some countries specifying maximal or exact levels, while others lack national standards. Data on fortified foods are limited in several Central and Southern European countries. Modeling indicates that multi-vehicle fortification is more effective than single-vehicle approaches, safely increasing population intakes while reducing deficiency prevalence. Conclusions: Vitamin D fortification policies across Europe are highly heterogeneous. Most countries rely on voluntary approaches, which provide limited coverage. Strengthening policy through mandatory and well-coordinated multi-vehicle strategies, informed by modeling and population-based studies, can improve vitamin D intake, reduce deficiency prevalence, and enhance health equity.

## 1. Introduction

Vitamin D is essential for human health, particularly for calcium and phosphate metabolism and the maintenance of bone integrity. Beyond its skeletal functions, observational evidence increasingly links low serum vitamin D levels to diverse extra-skeletal outcomes. These include effects on immune, metabolic, cardiovascular, and neurocognitive functions, though definitive causal relationships remain elusive [1].

For most individuals, vitamin D is produced in the skin after ultraviolet B exposure, while dietary intake and supplementation provide complementary sources. Endogenous synthesis varies substantially with latitude, season, age, skin pigmentation, clothing, and time spent outdoors. Natural dietary sources are limited, mainly to animal-based foods such as fatty fish, egg yolks, and liver, with minor contributions from plant-derived or fungal sources [2].

Vitamin D status is assessed by measuring serum 25-hydroxyvitamin D [25(OH)D], the principal circulating metabolite reflecting both dietary intake and endogenous synthesis [3]. Concentrations below 30 nmol/L (12 ng/mL) are typically defined as deficiency, while 30–50 nmol/L (12–20 ng/mL) indicates insufficiency for bone health [4]. Recent pooled estimates from large population-based studies, including a global analysis of 7.9 million participants from 308 studies, indicate that approximately 18% of individuals in Europe have serum 25(OH)D concentrations below 30 nmol/L, and approximately 53% fall below 50 nmol/L [5]. The prevalence of vitamin D deficiency varies substantially across European regions. Lower rates are observed in Northern Europe (generally <20–30% below 50 nmol/L), whereas higher prevalence is reported in Western, Central, and Southern Europe (30–60%), as summarized in a European position statement based on standardized datasets [6].

Certain population groups remain at particularly high risk. Vulnerable groups include older adults, individuals with darker skin, infants without supplementation, pregnant and lactating women, residents of high-latitude regions, and individuals with obesity or chronic diseases affecting vitamin D metabolism [7]. Given the limited natural dietary supply and seasonal constraints on dermal synthesis, food fortification has been recognized as a population-level strategy to improve vitamin D intake and reduce deficiency prevalence [8].

At a global level, vitamin D deficiency and insufficiency are prevalent across multiple regions. In the Middle East and parts of Asia (e.g., India and China), low serum 25(OH)D levels are very common despite abundant solar radiation. In contrast, vitamin D status is generally better in North America and Canada, where systematic fortification of staple foods such as milk and relatively higher supplement use have contributed to fewer cases of deficiency, although insufficiency persists. Poor to moderate vitamin D status is also common in African populations, likely reflecting cultural practices and skin pigmentation factors that limit effective dermal synthesis [9].

The effectiveness of fortification depends on the selection of widely consumed food vehicles and, where appropriate, the use of multiple vehicles to reach population groups with diverse dietary patterns. Evidence from systematic reviews and long-standing national fortification programs indicates that coordinated interventions can improve serum 25(OH)D concentrations and reduce deficiency prevalence at the population level [10].

In Europe, vitamin D policies are largely informed by reference values established by the European Food Safety Authority (EFSA), including the Adequate Intake (AI) and the Tolerable Upper Intake Level (UL), which provide the basis for safety assessments of fortification and supplementation. Typical fortification levels range from 1 to 5 µg per 100 g or 100 mL of product, depending on the vehicle, and are generally compatible with UL [11]. EFSA has established a UL of 100 µg/day for adults, 50 µg/day for children aged 1–10 years, and 25 µg/day for infants [12]. Despite this scientific and regulatory framework, fortification approaches across Europe remain heterogeneous, ranging from mandatory, centrally coordinated programs to voluntary, market-driven systems. This variability has important implications for coverage, equity, and public health impact.

This review provides a structured overview of vitamin D food fortification policies in Europe, aiming to (i) map regulatory frameworks and implementation models across EU Member States, EFTA countries, and the UK, (ii) summarize fortified food types and levels, and (iii) evaluate their potential to improve population-level vitamin D status.

## 2. Materials and Methods

This study was conducted as a document-based comparative review of national vitamin D food-fortification policies in Europe, supported by a targeted search of peer-reviewed literature for contextual interpretation.

Official legal acts and governmental documents regulating vitamin D food fortification were retrieved from public regulatory portals of all European Union (EU) Member States (*n* = 27), the European Free Trade Association (EFTA) countries (Norway, Iceland, Liechtenstein, and Switzerland; *n* = 4), and the United Kingdom.

For EU countries, the review was based on Regulation (EC) No 1925/2006 on the addition of vitamins, minerals, and certain other substances to foods [13], along with relevant national implementation measures where available. For EFTA countries and the UK, corresponding national legislation and official regulatory guidance were examined.

Searches of regulatory portals used targeted keywords related to vitamin D fortification. These included combinations of terms such as vitamin D, cholecalciferol, ergocalciferol, fortification, enrichment, addition of vitamins, and maximum levels, as well as their equivalents in relevant national languages. Search terms in national languages were generated using publicly available translation tools and cross-checked against terminology used on official national authority websites. Examples of the search terms used are provided in Appendix A, Table A2. Only legally binding or officially applicable documents in force at the time of the search, with consolidated versions where available, were considered.

Infant and follow-on formulas were excluded, as they are regulated under Regulation (EU) 2016/127, supplementing Regulation (EU) No 609/2013, and are subject to specific mandatory compositional requirements distinct from general food fortification frameworks [14,15].

For each country, regulatory information was extracted and summarized in regional comparative tables (Northern, Western, Central, and Southern Europe). The following variables were extracted:

- country;
- competent national authority or issuing body;
- legal basis or regulatory framework;
- fortification-policy status;
- permitted food categories (fortification vehicles);
- legally specified fortification levels or maximum permitted limits, where applicable;
- implementation notes, including documentation gaps, notification systems, positive-list systems, or market-driven practices.

When numerical levels or limits were not defined in the legal text, they were recorded as “not specified.”

Food-based dietary guidelines (FBDGs) were reviewed solely for contextual reference and were not included in the primary regulatory analysis; their key characteristics (issuing body, title, and year of the most recent edition/update) were summarized separately in Appendix A (Table A1).

To support interpretation of the regulatory findings, targeted searches of peer-reviewed literature were conducted in PubMed and Scopus. This secondary search focused primarily on European countries and provided context and interpretation, complementing primary legal and governmental sources. Search terms included combinations of “vitamin D”, “food fortification”, “fortification policy”, “micronutrient fortification policies”, “population vitamin D status”, “modeling”, “implementation”, and country names. Studies were considered eligible if they were peer-reviewed and relevant to vitamin D fortification policy, implementation, population vitamin D status, modeling of fortification strategies, or comparative policy context. Non-official sources, commercial materials, duplicate records, and studies without direct relevance to policy interpretation were excluded. All sources were accessed between December 2025 and March 2026.

## 3. Results

**Figure 1:**
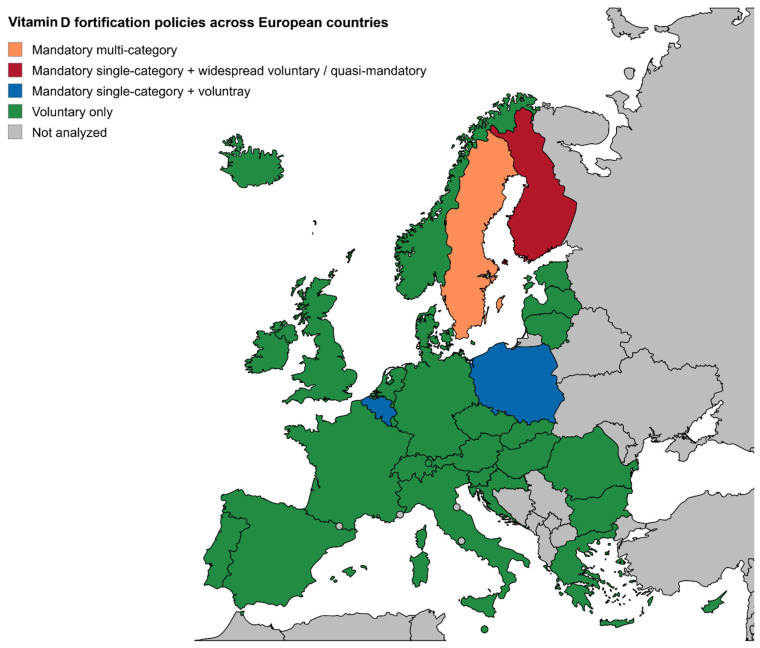
Vitamin D fortification policies across European countries. Countries are categorized according to the type of fortification policy: mandatory multi-category (orange), mandatory single-category with widespread voluntary or quasi-mandatory fortification (red), mandatory single-category with voluntary fortification (blue), voluntary fortification only (green), and not analyzed (gray). Sweden applies mandatory fortification across multiple food categories (including milk, fermented dairy products, and fat spreads). Finland represents a mixed system with mandatory fortification of skim milk combined with extensive voluntary fortification of other foods. Poland and Belgium maintain mandatory fortification limited to margarine and certain edible fats, with additional voluntary fortification. Most other EU and EFTA countries, as well as the United Kingdom, rely on voluntary fortification only, without specific national fortification policies.

Vitamin D food fortification policies across Europe are characterized by substantial heterogeneity in regulatory approach, eligible food categories, and the specification of fortification levels. Results of this study are presented by geographic regions (Northern, Western, Central, and Southern Europe) to reflect similarities in latitude, sun exposure, dietary habits, and food availability, which influence the context and effectiveness of vitamin D fortification strategies. This regional grouping also allows meaningful comparisons across countries with similar population-level risk factors. Additionally, Figure 1 presents the classification of countries according to mandatory or voluntary fortification.

National food-based dietary guidelines (FBDGs), presented in Appendix A, Table A1, show considerable variation across Europe in how vitamin D is addressed. In some countries, such as Finland and Sweden, FBDGs explicitly include vitamin D and reference fortified foods or population-level measures. In most other countries, vitamin D is mentioned indirectly through recommended food groups (e.g., dairy, fish) or general nutrient adequacy, without specific guidance on fortification. Several countries (e.g., Germany, Austria, Croatia, Romania) currently lack publicly available or updated FBDG documents. These differences in FBDGs reflect variation in the institutional frameworks and formal policy context that support vitamin D fortification initiatives, providing a backdrop for the heterogeneity observed in national fortification strategies.

A supplementary table presenting examples of the search terms used to identify national fortification-policy documents in English and relevant national languages is provided (Appendix A, Table A2).

With respect to fortification policies (Table 1, Table 2, Table 3 and Table 4), only four out of 32 (12.5%) countries analyzed (Finland, Sweden, Belgium, and Poland) have implemented mandatory vitamin D fortification for at least some foods. Finland mandates fortification of homogenized skimmed milk; Sweden applies mandatory fortification to defined categories of milk, fermented milk products, plant-based alternatives, and fat spreads; Belgium and Poland mandate fortification of margarine and selected edible fats. In all other countries reviewed, vitamin D fortification is permitted on a voluntary basis under Regulation (EC) No 1925/2006. In EEA countries such as Norway, Iceland, and Liechtenstein, fortification is permitted through national legislation implementing the same Regulation. In non-EEA/EFTA countries such as Switzerland, fortification is allowed under national legislation. In the United Kingdom, fortification policy is permitted under national legislation, historically based on EU legislation. However, fortification remains voluntary and market-driven, with implementation largely dependent on manufacturers and national guidance.

The range of foods eligible for vitamin D fortification differs markedly between countries. In Northern Europe, fortification extends beyond fat spreads to include fluid milk, fermented dairy products, and plant-based alternatives (Table 1), whereas in most Central, Western, and Southern European countries, fortification is concentrated primarily in margarines, breakfast cereals, selected dairy products, and plant-based beverages (Table 2, Table 3 and Table 4).

The specification of vitamin D levels in fortified foods varies considerably across countries (Table 1, Table 2, Table 3 and Table 4). While some countries, particularly in Northern Europe (e.g., Sweden, Finland, Norway, Germany, Belgium, and Poland), define specific fortification levels or maximum permitted amounts for certain food categories, others do not provide nationally specified limits in the available documentation. In these cases, vitamin D addition is generally guided by broader EU regulations or internationally recognized recommendations. Consequently, Table 1, Table 2, Table 3 and Table 4 do not report fortification limits for all countries.

## 4. Discussion

Vitamin D deficiency remains a persistent public health challenge throughout Europe, despite clear evidence that adequate status can be attained through sunlight exposure, diet, food fortification, and supplementation. The heterogeneity in national vitamin D fortification strategies reflects regulatory divergence across countries, including EU Member States operating under the flexible framework of Regulation (EC) No 1925/2006, as well as non-EU countries governed by distinct national regulatory approaches. These differences in legal frameworks, policy design, and implementation contribute to substantial variability in vitamin D intake and status across Europe.

According to WHO guidelines, the choice between mandatory and voluntary fortification should be guided by the severity of the public health need and the intended population-level impact, with the overarching goal of reducing vitamin D deficiency at the population level. For vitamin D, both D2 and D3 (preferably in stabilized forms) can be used, with milk, dairy products, and margarines recognized as suitable vehicles, especially in regions with limited sunlight exposure [8,30]. Mandatory fortification is recommended when deficiency constitutes a significant public health problem requiring wide and predictable coverage, as it ensures more uniform exposure and is less dependent on consumer awareness or market demand. Voluntary fortification may be appropriate in lower-risk settings or where consumer choice is prioritized; however, its population impact is less predictable, as it depends on industry uptake and regular consumption by those at risk [8,30].

Under well-regulated conditions, voluntary approaches can achieve comparable benefits, particularly when fortified foods are widely consumed and supported by monitoring and consumer education. Consequently, population-level data on vitamin D intake and status are critical for assessing the effectiveness of fortification programs and informing evidence-based policy decisions, with modeling providing a complementary tool to predict potential impacts and optimize program design.

Evidence from the EU-funded ODIN project (2013–2017), conducted across 19 European countries, demonstrated that baseline vitamin D intakes in Europe are typically low (3–5 µg/day), substantially below the dietary reference intake of 10 µg/day, and that reliance on a single fortified food vehicle is insufficient. Using harmonized dietary intake data and modeling approaches, ODIN showed that a multi-vehicle strategy, including fortification of milk and cheese and biofortification of eggs and meat, can safely raise vitamin D intakes to recommended levels, reduce deficiency prevalence by approximately 40–45%, and prevent seasonal declines in serum 25(OH)D concentrations [31].

### 4.1. Practical Challenges in Implementing Vitamin D Food Fortification Policies

Vitamin D fortification programs represent a specific application of large-scale food fortification (LSFF), a public health strategy aimed at improving population micronutrient status [8]. Implementation of such programs involves regulatory, industrial, and population-level challenges related to governance frameworks, industry capacity, consumer acceptance, dietary practices, population heterogeneity, and the availability of robust monitoring systems (Table 5) [8,30].

Country-specific studies illustrate these barriers in practice. In Belgium, regulatory constraints and modeling based on representative food consumption data influenced the design and potential effectiveness of fortification strategies [32]. In Romania, achieving target vitamin D intake through fortified foods was limited by both industry uptake and variability in consumption patterns [33]. Analyses of branded food composition databases in Slovenia also revealed gaps in implementation and monitoring, highlighting the importance of reliable data for evaluating real-world fortification practices [34].

Addressing these challenges is essential for designing effective vitamin D fortification strategies and may partly explain variations in national policies across Europe.

EFSA provides evidence-based Dietary Reference Values (DRVs), including AI and UL, which serve as a scientific benchmark for nutrient intake and safety assessments within the European Union [35]. However, implementing a uniform fortification policy across Europe remains challenging. Differences in regulatory frameworks, food production systems, dietary patterns, sun exposure, and population risk profiles limit the feasibility and effectiveness of a single EU-wide approach. Consequently, while harmonization can improve consistency in public health guidance, countries often adapt strategies to local circumstances, balancing scientific recommendations with practical, economic, and cultural considerations [36,37]. These factors help explain why national vitamin D policies may deviate from EFSA guidance despite a broadly shared scientific framework.

### 4.2. Regional Variation in Vitamin D Fortification Policies Across Europe

Across Europe, vitamin D food fortification practices and regulatory frameworks vary widely in terms of eligible food categories and permitted fortification levels. To better illustrate these differences, the following sections discuss regional patterns in vitamin D fortification policies across Northern, Western, Central, and Southern Europe.

#### 4.2.1. Northern Europe

In Northern Europe, national food-based dietary guidelines are based on the shared scientific framework of the Nordic Nutrition Recommendations (NNR2023) [38], which provide evidence-based nutrient reference values and form the foundation for country-specific guidance. However, the NNR are distinct from direct policy instruments such as fortification regulation, illustrating that coordinated scientific guidance does not automatically translate into uniform fortification policies across Nordic countries.

Finland operates one of the most comprehensive fortification systems in Europe. Since 2003, fluid milk products and fat spreads have been fortified with vitamin D3, with levels doubled in 2010 [39,40]. Although vitamin D fortification was initially implemented on a voluntary basis and widely adopted by industry, Finland introduced mandatory fortification of homogenized skimmed milk in 2016, while other milk products and fat spreads remain subject to voluntary fortification recommendations [16]. In the short term, wintertime serum 25(OH)D concentrations among young Finnish men increased by approximately 50%, with a marked reduction in deficiency prevalence [41]. Long-term population-based analyses confirmed these gains, showing an increase in mean serum 25(OH)D from ~48 to ~65 nmol/L between 2000 and 2011 and a decline in the proportion below 50 nmol/L from over 50% to less than 10% [42]. Fortified milk and spreads now contribute 29–39% of total dietary vitamin D intake, making fortified foods the dominant dietary sources of vitamin D alongside fish dishes [43]. Modeling studies indicate that current fortification levels maintain wide safety margins and that modest increases of approximately 1.2–1.5 µg/100 kcal could further enhance vitamin D adequacy without approaching the tolerable upper intake level [44]. Nevertheless, suboptimal status persists in certain subgroups, notably adolescents and low consumers of fortified products [39,45].

Sweden similarly operates a structured fortification policy. Mandatory vitamin D3 fortification initially covered selected milk products and spreads [46]. In 2018, the policy was expanded to include fermented milk products, eligible yogurts, and a broad range of plant-based drinks, with higher fortification levels [18]. Evidence indicates that this expansion increased population coverage and the potential for improved intakes, including in vulnerable groups, while remaining safely below upper intake levels [47,48].

Norway, Denmark and Iceland rely on voluntary fortification within a robust regulatory framework. Norway’s model uses a positive-list system, covering mainly margarine and other spreadable fats, selected milk products, and certain dairy alternatives, but the overall scope remains limited, and fortified foods contribute only modestly to intake [39,49]. Voluntary vitamin D fortification is regulated under national legislation (Regulation FOR-2010-02-26-247) [21], implemented within the framework of the European Economic Area (EEA) and aligned with EU provisions on the addition of vitamins and minerals to foods (Regulation (EC) 1925/2006). The national regulation specifies approved food categories and maximum fortification levels, yet few low-fat dairy products are fortified, and plant-based drinks, cereals, and other staple foods are rarely included. Consequently, a substantial proportion of the population does not achieve the recommended intake of 10 µg/day from diet alone [39,49]. Groups at elevated risk of inadequate intake include adolescents, older adults with low fish consumption, individuals with limited supplement use, and certain immigrant populations. Norway thus exemplifies a model in which robust, evidence-based dietary guidance coexists with a fortification strategy of limited scope, highlighting both the strengths of coordinated nutrition policy and the potential for broader fortification measures to improve population-level vitamin D status, as observed in other Nordic countries such as Finland and Sweden [39,50]. Denmark allows fortification mainly of fat spreads, with additional foods authorized on a case-by-case basis by the National Food Institute. Danish modeling studies and intervention trials suggest that expanding fortification to multiple staple foods, such as milk and bread, could raise population intakes to recommended levels while remaining safely below upper intake limits [51,52]. Until such measures are implemented, dietary adequacy largely depends on supplementation and consumption of naturally vitamin D–rich foods [53]. In Iceland, dietary vitamin D relies heavily on traditional sources, particularly fish and cod liver oil, while fortification of domestic products is neither mandatory nor universal, limited to most fat spreads and some fluid milk, with certain imported foods also fortified [39,54].

The Baltic states face similar seasonal challenges but lack comparable fortification evidence. Vitamin D addition to foods is permitted under EU law, but no mandatory programs exist, and data on voluntary fortification are scarce, leaving the contribution of fortified foods unclear.

#### 4.2.2. Western Europe

Western European countries generally base dietary reference values on EFSA guidance, but fortification is regulated nationally. As a result, fortification remains largely voluntary and market-driven, leading to substantial variation in approaches and limited coverage of key food categories.

Belgium combines mandatory fortification of margarine and edible fats at legally specified levels (6.25–7.5 µg/100 g) with voluntary fortification in other food categories, thereby retaining a regulated population-wide vehicle for vitamin D delivery [23]. However, population vitamin D intake remains below recommended levels, and fortified foods and supplements only marginally improve median intakes [32]. Modeling studies have therefore been proposed to identify optimal combinations of food vehicles and fortification levels [55].

In the UK and Ireland, voluntary fortification has supported specific product niches but has not delivered broad population coverage. The UK’s historical mandatory margarine fortification legacy (abolished in 2013) has not translated into a structured program for staple foods, and deficiency remains prevalent in several at-risk groups, particularly among ethnic minority groups, pregnant women, adolescents and individuals with limited sunlight exposure [56,57]. Ireland similarly lacks a coordinated national strategy to scale up population intake [58,59].

The Netherlands, France, Switzerland, and Austria also demonstrate that voluntary, market-driven systems may lead to fragmented fortification concentrated in a limited set of product categories, without explicit public health objectives or coordinated strategies [60,61]. This approach may perpetuate inequalities, as vulnerable groups with low supplement use or limited access to fortified foods are less likely to benefit.

Germany represents a distinct case where a regulatory framework exists, but historical legal constraints and conservative risk models limit fortification scope. Current legislation restricts vitamin D addition to a narrow range of foods, and the Federal Institute for Risk Assessment (BfR), as the national scientific advisory body, sets relatively low maximum levels based on risk assessment. Consequently, fortification remains limited, which may contribute to persistent seasonal insufficiency [11,27,62].

#### 4.2.3. Central Europe

Central European countries largely follow the broader EU pattern of voluntary, market-driven vitamin D fortification, with limited regulation and no coordinated public-health strategy. Multi-country modeling suggests that current voluntary approaches are insufficient to close the gap between actual and recommended intakes, and deficiency remains common among older adults and other at-risk subgroups [60].

Poland represents a cautious, stepwise model. Mandatory fortification of spreadable fats has been in place since 2010 and was reaffirmed in 2024, maintaining fats (excluding milk fats) as the sole mandatory vehicle at levels up to 7.5 µg/100 g [28,63]. Despite this long-standing regulatory measure, vitamin D intake and status remain suboptimal across age groups, with dietary intakes frequently far below recommendations and widespread deficiency documented in national assessments [64]. The 2024 regulation signals renewed recognition of fortification as a public-health instrument and improves regulatory coherence, yet the continued restriction of mandatory fortification to a single food category limits its potential population reach [28]. Consequently, supplementation remains a central strategy for infants, adolescents, older adults and individuals with limited sun exposure.

In Bulgaria, Hungary, Slovakia, the Czech Republic, Romania, and Slovenia, vitamin D fortification is legally permitted but remains voluntary and unevenly implemented. Fortification is limited to selected product categories and is poorly documented at the national level. Consequently, fortification remains fragmented and is unlikely to function as an effective population-wide public health intervention [33,34,65].

#### 4.2.4. Southern Europe

A major contributing factor is the limited impact of food fortification policies. Unlike Northern Europe, where fortification of selected foods (e.g., milk and spreads) is systematically implemented, Southern European countries rely on voluntary rather than mandatory fortification, which alone is insufficient to ensure population-level adequacy [60]. Population-based studies indicate that mean serum 25(OH)D concentrations are often below 50 nmol/L, with a substantial proportion of participants exhibiting vitamin D deficiency, based on data collected in different seasons [66]. This apparent discrepancy between abundant sunlight and widespread deficiency, referred to as the “Mediterranean paradox,” reflects the fact that effective cutaneous vitamin D synthesis depends not only on sunlight availability but also on actual skin exposure to UVB. Behavioral and environmental factors, including clothing, indoor living promoted by urbanization, sedentary lifestyles, high ambient temperatures, sunscreen use, and seasonality, significantly limit UVB-induced vitamin D production, particularly during peak sun hours [6]. Aging populations are especially vulnerable due to a diminished capacity for cutaneous vitamin D synthesis combined with lower outdoor exposure [67]. In addition, lifestyle changes and the westernization of dietary patterns, particularly among younger populations, have reduced adherence to traditional Mediterranean diets, historically rich in vitamin D sources, resulting in insufficient dietary intake [68,69].

Despite documented deficiencies, most Southern European FBDGs address vitamin D only indirectly, through general nutrient adequacy recommendations, and do not provide explicit guidance on supplementation or fortified foods, unlike higher-latitude countries such as Finland or Sweden (Table A1). FBDG development in the region prioritizes general diet-health relationships, while geographical, behavioral, and dietary factors highlight a gap in current guidelines and the need for context-specific nutrition policies to ensure adequate vitamin D status.

### 4.3. Monitoring and Oversight of Large-Scale Food Fortification (LSFF) Programs

The diversity in fortification strategies across Europe is accompanied by corresponding differences in monitoring and enforcement approaches, as described in Section 4.2.

**Figure 2:**
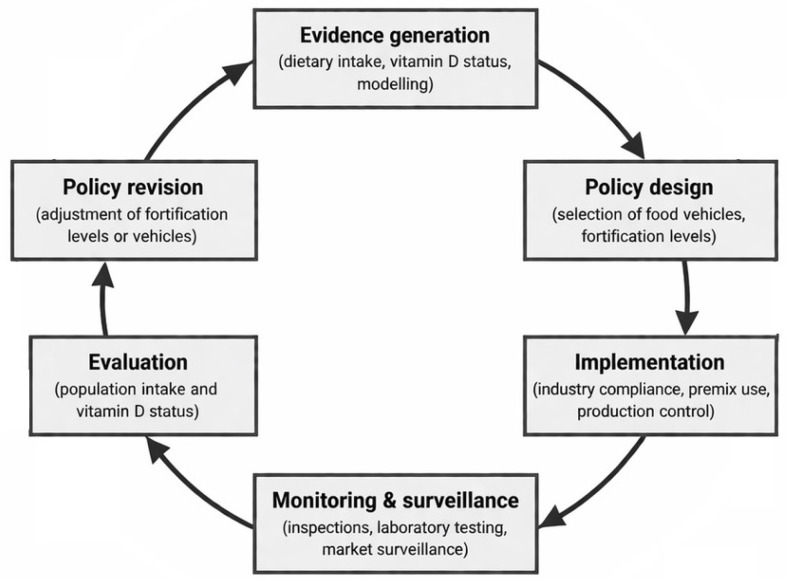
LSFF policy cycle for vitamin D fortification. Key stages include evidence generation, policy design, implementation, monitoring and surveillance, evaluation, and policy revision. Monitoring and oversight operate across multiple stages to ensure compliance, effectiveness, and safety.

LSFF programs rely on robust monitoring and oversight to ensure compliance, effectiveness, and safety. These functions are integral components of the broader LSFF policy cycle (Figure 2), spanning evidence generation, policy design, implementation, and evaluation. Policy oversight involves multiple stakeholders, including ministries of health, agriculture, and trade; food producers and importers; non-governmental organizations; and consumer groups [70,71].

Once fortification strategies and technical standards are established, regulatory frameworks define which foods should be fortified, the permitted micronutrients and levels, and the institutions responsible for supervision. Implementation requires systems for enterprise registration and licensing, including controls over production, import, and distribution, as well as inspections to verify compliance with regulatory requirements [70]. Monitoring generally combines compliance inspections, laboratory testing, and targeted surveillance of higher-risk enterprises. Population-level monitoring, such as dietary intake and biomarker assessments, provides evidence on program coverage and effectiveness [71].

In countries with mandatory vitamin D fortification, monitoring is structured and systematic. In Finland, the Finnish Food Authority provides detailed guidance for controlling fortified foods, including enterprise registration, documentation review, inspections, laboratory testing, and labeling verification. Producers are required to notify authorities and maintain internal control systems to ensure compliance with regulatory requirements [72]. In Sweden, the Swedish National Food Agency conducts systematic monitoring of mandatory products through inspections, laboratory analyses, and verification of compliance, while voluntary fortification is assessed through risk-based controls focused on labeling, documentation, and nutrient levels [73].

In Belgium, monitoring and enforcement of fortified foods are carried out by the Federal Agency for the Safety of the Food Chain (FASFC), which conducts official controls, including inspections and sampling, across the entire food chain to ensure compliance with national and EU food legislation [74]. This system is complemented by a nutrivigilance framework introduced in 2024, which enables the detection and evaluation of adverse effects associated with vitamin- and mineral-enriched foods, thereby strengthening post-market surveillance [75]. In Poland, oversight is implemented within the general food safety system under the Chief Sanitary Inspectorate. A key component is the mandatory notification of fortified products before market placement, supported by a central register and routine inspections to verify composition, labeling, and compliance [76].

In countries with voluntary vitamin D fortification, monitoring is more complex because products are not subject to mandatory nutrient standards. Oversight relies primarily on general food safety systems, including market surveillance, labeling verification, review of production documentation, and targeted laboratory testing to assess compliance with declared nutrient levels [71,77]. As a result, monitoring in voluntary systems is generally less standardized and more fragmented than in mandatory programs, with greater reliance on post-market control rather than systematic pre-defined compliance frameworks. Although population-level monitoring may complement these activities, the absence of mandatory reporting requirements and variability in market participation limit the ability to conduct systematic evaluation.

## 5. Study Limitations

This review has several limitations. The analysis focused primarily on official regulatory documents and publicly available policy information, which may not fully capture market practices or the actual availability of fortified foods. In addition, the implementation of voluntary fortification varies across countries and may not be comprehensively documented in national legislation. Finally, the study did not quantitatively assess dietary intake data or population vitamin D status, which may influence the real-world impact of fortification policies.

## 6. Conclusions and Future Perspectives

Most European countries rely predominantly on voluntary, market-driven fortification, resulting in low coverage, limited diversity of fortified foods, and modest contributions to overall vitamin D intake.

Consequently, prevention depends largely on individual supplementation, which is unevenly adopted and leaves high-risk groups underprotected. In contrast, centrally coordinated mandatory or near-mandatory programs in Finland and Sweden achieve high population coverage, measurable improvements in vitamin D status, and demonstrate the effectiveness of multi-vehicle strategies, including fortification of milk, cheese, eggs, and meat.

Future efforts should prioritize robust monitoring and evaluation systems. These should integrate regulatory oversight at production and retail levels with population-based assessments of program coverage, dietary intake, and vitamin D status. In some European countries, data on fortified foods and the population’s vitamin D status remain limited or incomplete. Expanding population-based studies and applying modeling approaches, such as those used in the ODIN project, can inform multi-vehicle fortification strategies, estimate population-level impacts, and guide context-specific policy decisions. Enhancing public awareness and promoting dietary habits that include fortified foods are essential to ensure adequate intake, particularly among vulnerable groups. Coordinated, evidence-informed approaches can improve equity, enable cross-country comparisons, and support potential alignment of vitamin D fortification policies across Europe.

## Figures and Tables

**Table 1:** Vitamin D fortification policies and legal frameworks in Northern European countries.

Country	National Authority	Fortified Foods & Typical Vitamin D Content	Legal Basis/Regulation	Notes/Comments
Finland	Ministry of Agriculture and Forestry (food legislation); Finnish Food Authority (Ruokavirasto) (food control and enforcement)	Homogenized skimmed milk: ~1 µg/100 mL; Fat spreads & margarine: ~20 µg/100 g; Plant-based milk and selected bread/cereals: levels vary by product or brand	Regulation (EC) 1925/2006 on addition of vitamins and minerals [13] National Decree 754/2016 (mandatory fortification of homogenized skimmed milk) [16]; National Decree 917/2002 (framework for voluntary addition of vitamins and other substances to foods) [17];	Mandatory fortification applies only to homogenized skimmed milk. Fortification of other food categories is voluntary or market-driven but follows national vitamin D fortification recommendations issued by the Finnish National Nutrition Council
Sweden	Ministry of Rural Affairs and Infrastructure (food legislation); Swedish National Food Agency (Livsmedelsverket) (food control and enforcement)	Milk and milk alternatives (≤3% fat): 0.95–1.10 µg/100 g; fermented milk & fermented plant-based alternatives (≤3% fat): 0.75–1.10 µg/100 g; Fat spreads & margarine: 19.5–21.0 µg/100 g	Regulation (EC) 1925/2006 on addition of vitamins and minerals [13] Swedish Food Agency regulations (LIVSFS 2018:5) [18];	Mandatory fortification applies to specified categories of milk, fermented milk, plant-based milk alternatives, and fat spreads/margarines, as defined in LIVSFS 2018:5. Fortification of other food categories is voluntary or market-driven (levels not set by regulation)
Denmark	Ministry of Food, Agriculture and Fisheries (food legislation); Danish Veterinary and Food Administration (Fødevarestyrelsen) (food control and enforcement)	Selected food categories (no nationally specified product list)	Regulation (EC) 1925/2006 on addition of vitamins and minerals [13]; Danish Veterinary and Food Administration (DVFA) guidance on fortified foods [19].	Voluntary/market-driven (levels not set by regulation); examples of fortified foods include fat spreads and margarine, plant-based beverages, and fortified cereals (market practice, not defined by regulation)
Norway	Ministry of Agriculture and Food (food legislation); Norwegian Food Safety Authority (Mattilsynet) (food control and enforcement)	Maximum levels (µg/100 g or µg/100 mL): spreadable fats/margarine 20, butter 10, cheese 2.7–4.1, milk (all types sold directly to consumers/fluid milk) 1.0, milk-based beverages 1.9, fermented milk products 2.9, dairy analogs 1.5, fruit/vegetable blend beverages 2.5, breakfast cereals/bakery products 5	National regulation with positive lists and notification [20]; Regulation FOR-2010-02-26-247 on the addition of vitamins, minerals and certain substances to foodstuffs [21]	Voluntary fortification; products must be on positive list and addition notified to Mattilsynet
Iceland	Ministry of Food, Agriculture and Fisheries (food legislation); Icelandic Food and Veterinary Authority (MAST) (food control and enforcement)	Selected food categories (no nationally specified product list)	Regulation (EC) No 1925/2006 on addition of vitamins and minerals [13] implemented in Iceland via Regulation 327/2010 [22]	Voluntary/market-driven (levels not set by regulation); examples of fortified foods include dairy products, plant-based beverages, spreads, and selected cereals (market practice, not defined by regulation)
Latvia	Ministry of Agriculture (food legislation); Food and Veterinary Service (PVD) (food control and enforcement)	NA	Regulation (EC) 1925/2006 on addition of vitamins and minerals [13]	Vitamin D addition is permitted under EU law; no mandatory fortification exists, and no official data on voluntarily fortified products are available
Estonia	Ministry of Regional Affairs and Agriculture (food legislation); Estonian Agriculture and Food Board (Põllumajandus-ja Toiduamet) (food control and enforcement)	NA	Regulation (EC) 1925/2006 on addition of vitamins and minerals [13]	Vitamin D addition is permitted under EU law; no mandatory fortification exists, and no official data on voluntarily fortified products are available
Lithuania	Ministry of Agriculture (food legislation); Lithuanian State Food and Veterinary Service (VMVT) (food control and enforcement)	NA	Regulation (EC) 1925/2006 on addition of vitamins and minerals [13]	Vitamin D addition is permitted under EU law; no mandatory fortification exists, and no official data on voluntarily fortified products are available

NA—not available (no publicly accessible data).

**Table 2:** Vitamin D fortification policies and legal frameworks in Western European countries.

Country	National Authority	Fortified Foods & Typical Vitamin D Content	Legal Basis/Regulation	Notes/Comments
Belgium	Federal Public Service Health, Food Chain Safety and Environment (FPS Health) (food legislation); Federal Agency for the Safety of the Food Chain (FASFC/AFSCA) (food control and enforcement)	Margarine, edible fats: 6.25–7.5 µg/100 g	Regulation (EC) 1925/2006 on addition of vitamins and minerals [13]; Royal Decree of 2 October 1980 on the manufacture and marketing of margarine and edible fats [23].	Mandatory vitamin D fortification applies to margarine and certain edible fats. Fortification of other food categories is voluntary; examples of fortified foods include milk, milk substitutes, dairy desserts, cereals, biscuits, chocolate powder, and fruit juices
United Kingdom	Department of Health and Social Care (food legislation); Food Standards Agency (FSA, England, Wales, Northern Ireland); Food Standards Scotland (FSS, Scotland) (food control and enforcement)	Selected food categories (no nationally specified product list)	Historically based on Regulation (EC) 1925/2006 on addition of vitamins and minerals [13]	Fortification is currently voluntary and market-driven (levels not set by regulation); examples of fortified foods include fat spreads, ready-to-eat breakfast cereals, dried and evaporated milks, and plant-based milk alternatives (market practice, not defined by regulation).
Ireland	Department of Health (food legislation); Food Safety Authority of Ireland (FSAI) (food control and enforcement)	Selected food categories (no nationally specified product list)	Regulation (EC) 1925/2006 on addition of vitamins and minerals [13] implemented by national guidance S.I. No. 376/2017 issued by FSAI [24]	Voluntary/market-driven (levels not set by regulation); examples of fortified foods include selected fluid milk and milk-based drinks, breakfast cereals, fat spreads (margarines), and certain plant-based alternatives (market practice, not defined by regulation)
The Netherlands	Ministry of Health, Welfare and Sport (food legislation); Netherlands Food and Consumer Product Safety Authority (NVWA) (food control and enforcement)	Selected food categories (no nationally specified product list); National exemption allows vitamin D addition up to 4.5 µg/100 kcal in selected products	Regulation (EC) 1925/2006 on addition of vitamins and minerals [13]; National exemption Warenwetregeling vrijstelling toevoeging foliumzuur en vitamine D (exemption allowing vitamin D addition up to 4.5 µg/100 kcal) [25]	Voluntary/market-driven (levels not set by regulation); examples of fortified foods include margarines and other plant-based fats, dairy substitutes (plant-based milks), and some non-alcoholic beverages (market practice, not defined by regulation)
France	French Ministry of Agriculture and Food Sovereignty (food legislation); Directorate General for Competition, Consumer Affairs and Fraud Control (DGCCRF) (food control and enforcement)	Selected food categories (no nationally specified product list)	Regulation (EC) 1925/2006 on addition of vitamins and minerals [13]	Voluntary/market-driven (levels not set by regulation); examples of fortified foods include selected dairy products, certain margarines, fat spreads, breakfast cereals, and vegetable oils (market practice, not defined by regulation)
Switzerland	Swiss Federal Department of Home Affairs (FDHA) (food legislation); Federal Food Safety and Veterinary Office (FSVO) (food control and enforcement)	Selected food categories (no nationally specified product list)	Ordinance on the Addition of Vitamins, Minerals and Other Substances to Foodstuffs (AVMO) [26]	Voluntary/market-driven (levels not set by regulation); examples of fortified foods include margarine, butter, dairy products, certain oils, juices, and breakfast cereals (market practice, not defined by regulation); Maximum levels exist as guidance derived from the Maximum Level Model based on tolerable daily intake and typical consumption per product category, not explicitly listed in AVMO Annex 1.
Germany	Department of Health and Social Care (food legislation); Food Standards Agency (FSA, England, Wales, Northern Ireland); Food Standards Scotland (FSS, Scotland) (food control and enforcement)	Maximum vitamin D levels (µg/100 g or µg/100 mL): milk and dairy products, including cheese 1.5, bread and cereals (excluding pastries) 5.0, spreadable fats and cooking oil 7.5, other foods—no addition	Regulation (EC) 1925/2006 on addition of vitamins and minerals [13]; BfR national risk-based guidance for maximum vitamin D levels [27]	Voluntary/market-driven; Maximum vitamin D levels are derived using a regulatory fortification model, with levels expressed as up to 20 µg per daily serving of selected foods.
Austria	Department of Health (food legislation); Food Safety Authority of Ireland (FSAI) (food control and enforcement)	Selected food categories (no nationally specified product list)	Regulation (EC) 1925/2006 on addition of vitamins and minerals [13]	Voluntary/market-driven (levels not set by regulation); examples of fortified foods include margarines, plant-based beverages, and dairy alternatives (market practice, not defined by regulation)
Luxembourg	Luxembourg Veterinary and Food Administration (ALVA)—national food-chain control authority	NA	Regulation (EC) 1925/2006 on addition of vitamins and minerals [13]	Vitamin D addition is permitted under EU law; no mandatory fortification exists, and no official data on voluntarily fortified products are available
Liechtenstein	Food Control and Veterinary Office—national authority for food control	NA	Swiss food legislation applies; Ordinance on the Addition of Vitamins, Minerals and Other Substances to Foodstuffs (AVMO) [26]	Voluntary/market-driven (levels not set by regulation); no official data on voluntarily fortified products are available

NA—not available (no publicly accessible data).

**Table 3:** Vitamin D fortification policies and legal frameworks in Central European countries.

Country	National Authority	Fortified Foods & Typical Vitamin D Content	Legal Basis/Regulation	Notes/Comments
Poland	Ministry of Health (food legislation); Chief Sanitary Inspectorate (GIS) (food control and enforcement)	Spreadable fats: maximum level 7.5 µg/100 g;	Regulation (EC) 1925/2006 on addition of vitamins and minerals [13] Regulation of the Minister of Health of 13 March 2024 on Substances Added to Food for Fortification Purposes [28];	Mandatory fortification limited to spreadable fats. Fortification of other food categories is voluntary/market-driven (levels not set by regulation); examples of fortified foods include cereals, dairy products, and plant-based milk alternatives
Bulgaria	Ministry of Health (food legislation); Bulgarian Food Safety Agency (BFSA) (food control and enforcement)	NA	Regulation (EC) 1925/2006 on addition of vitamins and minerals [13]	Vitamin D addition is permitted under EU law; no mandatory fortification exists, and no official data on voluntarily fortified products are available
Czech Republic	Ministry of Health (food legislation); State Agricultural and Food Inspection Authority (SZPI) (food control and enforcement)	Selected food categories (no nationally specified product list)	Regulation (EC) 1925/2006 on addition of vitamins and minerals [13]	Voluntary/market-driven (levels not set by regulation); examples of fortified foods include margarines, milk, plant-based beverages, dairy alternatives, some cocoa, and breakfast cereals (market practice, not defined by regulation)
Hungary	Ministry of Agriculture (food legislation); National Food Chain Safety Office (Nébih/NFCSO) (food control and enforcement)	NA	Regulation (EC) 1925/2006 on addition of vitamins and minerals [13]	Vitamin D addition is permitted under EU law; no mandatory fortification exists, and no official data on voluntarily fortified products are available
Romania	Ministry of Health (food legislation); National Sanitary Veterinary and Food Safety Authority (ANSVSA) (food control and enforcement)	Selected food categories (no nationally specified product list)	Regulation (EC) 1925/2006 on addition of vitamins and minerals [13]	Voluntary/market-driven (levels not set by regulation); examples of fortified foods include margarine, beverages, yogurt drinks, cereals, and soy products (market practice, not defined by regulation)
Slovakia	Ministry of Health (food legislation); Public Health Authority of the Slovak Republic (food control and enforcement	NA	Regulation (EC) 1925/2006 on addition of vitamins and minerals [13]	Vitamin D addition is permitted under EU law; no mandatory fortification exists, and no official data on voluntarily fortified products are available
Slovenia	Ministry of Health (food legislation); Administration of the Republic of Slovenia for Food Safety, Veterinary Sector and Plant Protection (AFSVSPP) (food control and enforcement)	Selected food categories (no nationally specified product list)	Regulation (EC) 1925/2006 on addition of vitamins and minerals [13]	Voluntary/market-driven (levels not set by regulation); examples of fortified foods include beverages, cereal and cereal products, edible oils, margarines, dairy and imitates (market practice, not defined by regulation)

NA—not available (no publicly accessible data).

**Table 4:** Vitamin D fortification policies and legal frameworks in selected counties of Southern Europe.

Country	National Authority	Fortified Foods & Typical Vitamin D Content	Legal Basis/Regulation	Notes/Comments
Greece	Ministry of Health (food legislation); Hellenic Food Authority (EFET) (food control and enforcement)	Selected food categories (no nationally specified product list)	Regulation (EC) 1925/2006 on addition of vitamins and minerals [13]	Voluntary/market-driven (levels not set by regulation); examples of fortified foods include dairy alternatives, breakfast cereals, juices/nectars, and margarines (market practice, not defined by regulation)
Croatia	Ministry of Health (food legislation); Croatian Agency for Agriculture and Food (HAPIH) (food control and enforcement)	Selected food categories (no nationally specified product list)	Regulation (EC) 1925/2006 on addition of vitamins and minerals [13]	Voluntary/market-driven (levels not set by regulation); examples of fortified foods include margarines and some dairy products (market practice, not defined by regulation)
Spain	Ministry of Health (food legislation); Spanish Agency for Food Safety and Nutrition (AESAN) (food control and enforcement)	Selected food categories (no nationally specified product list)	Regulation (EC) 1925/2006 on addition of vitamins and minerals [13]	Voluntary/market-driven (levels not set by regulation); examples of fortified foods include margarines, plant-based beverages, breakfast cereals, some dairy products, and juices (market practice, not defined by regulation)
Italy	Ministry of Health (food legislation and enforcement)	Selected food categories (no nationally specified product list)	Regulation (EC) 1925/2006 on addition of vitamins and minerals [13]	Voluntary/market-driven (levels not set by regulation); examples of fortified foods include milk, cereals, yogurt, and cheese (market practice, not defined by regulation)
Malta	Ministry for Health (food legislation); Environmental Health Directorate (food control and enforcement)	Selected food categories (no nationally specified product list)	Regulation (EC) 1925/2006 on addition of vitamins and minerals [13]	Voluntary/market-driven (levels not set by regulation); examples of fortified foods include yogurt, milk, and dairy products (market practice, not defined by regulation)
Portugal	Directorate-General for Health (DGS) (food legislation); Portuguese Food Safety Authority (ASAE) (food control and enforcement)	Selected food categories (no nationally specified product list)	Regulation (EC) 1925/2006 on addition of vitamins and minerals [13]	Voluntary/market-driven (levels not set by regulation); examples of fortified foods include breakfast cereals and fat spreads, yogurt, and milk (market practice, not defined by regulation)
Cyprus	Public Health Services, Ministry of Health; State General Laboratory (SGL)	NA	Regulation (EC) 1925/2006 on addition of vitamins and minerals [13]; Food (Control and Sale) Law of 1996 [29]	Vitamin D addition is permitted under EU law; no mandatory fortification exists, and no official data on voluntarily fortified products are available

NA—not available (no publicly accessible data).

**Table 5:** Practical challenges in implementing vitamin D food fortification policies.

Challenge Area	Description	Implications for Policy Design
Regulatory and legislative barriers	Clear legal frameworks are required to define permitted food vehicles, vitamin D forms, fortification levels, and labeling requirements	Differences in national food law and regulatory structures may lead to variation in the scope and strictness of fortification policies
Industry compliance and technical capacity	Fortification may require adjustments in production processes, formulations, and quality control to ensure consistent vitamin D levels	Countries with technologically advanced and centralized food industries may implement mandatory fortification more easily
Consumer acceptance and cultural factors	Cultural practices, dietary preferences, and nutrition awareness influence acceptance and consumption of fortified foods	Effective policies require selecting food vehicles that are widely consumed within the population
Economic feasibility and cost considerations	Fortification can increase production costs due to equipment, quality control, and staff training	Economic constraints may influence whether countries adopt mandatory or voluntary fortification, or rely more on supplementation
Population heterogeneity	Vitamin D requirements vary by age, skin pigmentation, lifestyle, and sun exposure	Policymakers must balance deficiency prevention with the risk of excessive intake when defining fortification levels
Limited population data and modeling evidence	Reliable data on dietary intake, vitamin D status, and food consumption patterns are not always available	In the absence of comprehensive data, countries may rely on modeling studies or adopt more cautious fortification strategies
Monitoring and evaluation systems	Effective programs require regulatory monitoring, production control, and periodic population surveys	Limited monitoring capacity may hinder implementation and evaluation of fortification policies

## Data Availability

Not applicable. No new data were created or analyzed in this study.

## Appendix A

**Table A1:** Food-based dietary guidelines (FBDGs) in selected European countries and their relevance to vitamin D policy.

Country	Issuing Body	Guideline Title (Year of Latest Edition/Update)	Content Characteristics
Finland	Finnish National Nutrition Council; Finnish Institute for Health and Welfare (THL)	*Sustainable Health from Food—National Nutrition Recommendations* (2024) [78]	Evidence-based, comprehensive quantitative guidance with food group targets and nutrient reference values; explicitly addresses vitamin D including fortified foods and population-level measures
Sweden	Swedish National Food Agency (Livsmedelsverket)	*Livsmedelsverkets generella kostråd för den vuxna befolkningen* (2025) [79]	Evidence-based, quantitative food group guidance with evidence-based background; vitamin D included in nutrient considerations, without explicit fortification policy
Denmark	Danish Veterinary and Food Administration (Fødevarestyrelsen)	*De officielle Kostråd—godt for sundhed og klima* (2024) [80]	Evidence-based, quantified food group guidance with practical messages; vitamin D addressed indirectly via recommended foods (e.g., fish, dairy)
Norway	Norwegian Directorate of Health (Helsedirektoratet)	*Kostråd for god helse og gode liv* (2024) [81]	Evidence-based, food-based dietary guidance with supporting materials; vitamin D embedded within broader dietary patterns, not as explicit fortification or supplementation policy
Iceland	Directorate of Health (Iceland)	*Food-Based Dietary Guidelines* (2025) [82]	Evidence-based, food-based dietary guidance with public-health context; vitamin D emphasized due to limited sunlight exposure.
Latvia	Centre for Disease Prevention and Control (SPKC)	*Healthy Eating Recommendations for Adults* (2020) [83]	Evidence-based, food-based guidance with recommended food groups and practical habits; vitamin D addressed indirectly via general nutrient adequacy
Estonia	National Institute for Health Development (TAI)	*Estonian Food-Based Dietary Guidelines* (2024)	NA
Lithuania	Institute of Hygiene (Higienos institutas)	*Healthy and Sustainable Dietary Recommendations* (2025) [84]	Evidence-based, food-based recommendations with health, sustainability, and quantitative focus; vitamin D addressed indirectly via overall nutrient adequacy
United Kingdom	UK Government	*The Eatwell Guide* (2016; updated access 2024) [85]	Evidence-based, plate-based food guidance for the general population; vitamin D addressed via public-health advice, not within the plate model
Ireland	Department of Health (Healthy Ireland)	*The Food Pyramid—Healthy Eating Guidelines* (2016) [86]	Evidence-based, portion-based food guidance with daily servings; vitamin D addressed indirectly via nutrient adequacy
Belgium	Superior Health Council of Belgium	*Food-based dietary guidelines for the Belgian population* (2019; update process 2025)	NA
The Netherlands	Health Council of the Netherlands	*Dutch dietary guidelines—protein sources and dietary patterns* (2025) [87]	Evidence-based food guidance on dietary patterns and quality; vitamin D addressed indirectly via nutrient adequacy
France	Santé publique France	*Guide alimentaire pour les adultes* (2019) [88]	Evidence-based, quantitative and qualitative food guidance; vitamin D addressed via nutrient adequacy, not explicit fortification policy
Switzerland	Federal Food Safety and Veterinary Office; Swiss Society for Nutrition	*Swiss dietary recommendations for adults* (2024) [89]	Evidence-based, portion-based food guidance with practical advice; vitamin D addressed indirectly via overall nutrient adequacy
Germany	German Nutrition Society (DGE)	*Ten guidelines for a wholesome diet* (2024)	NA
Austria	Austrian Agency for Health and Food Safety; Austrian Nutrition Society	*Austrian nutritional recommendations* (2025)	NA
Slovenia	National Institute of Public Health (NIJZ)	*Priporočila zdrave prehrane za odrasle* (2018) [90]	Evidence-based, implementation-oriented food guidance with practical advice; vitamin D addressed contextually, not as explicit fortification policy
Slovakia	Ministry of Health of the Slovak Republic	*Odporúčania pre stravovanie a výživu u dospelých* (2021) [91]	Evidence-based, food-based guidance on balanced dietary patterns; vitamin D addressed indirectly via general nutrition guidance
Croatia	Ministry of Health (Croatia)	*Dietary guidelines* (2002)	NA
Greece	Ministry of Health (Greece)	*National Dietary Guidelines for Adults* (2014) [92]	Evidence-based, food-based guidance for adults; vitamin D addressed within broader nutritional considerations, not as explicit fortification policy
Hungary	Hungarian Dietetic Association (MDOSZ)	*OKOSTÁNYÉR^®^—Hungarian dietary recommendations* (2021) [93]	Evidence-based, plate-based food guidance for adults; vitamin D addressed indirectly via general healthy diet principles
Italy	CREA—Council for Agricultural Research and Economics	*Linee guida per una sana alimentazione* (2018) [94]	Evidence-based, comprehensive food guidance with implementation support; vitamin D addressed via overall nutrient adequacy
Malta	Health Promotion and Disease Prevention Directorate, Ministry for Health	*Dietary Guidelines for Maltese Adults: Healthy Eating the Mediterranean Way* (2015) [95]	Evidence-based, Mediterranean-style food guidance with practical advice; vitamin D addressed contextually via nutrient adequacy
Poland	National Institute of Public Health NIH–NRI	*Healthy Eating Plate/Talerz Zdrowego Żywienia* (2020) [96]	Evidence-based, plate-based food guidance with quantitative and quality focus; vitamin D addressed indirectly, not as an explicit topic
Portugal	Direção-Geral da Saúde (DGS)	*Roda dos Alimentos* (2003; content updated 2024) [97]	Evidence-based, food-wheel guidance with quantitative focus; vitamin D addressed indirectly via overall dietary patterns
Romania	National Institute of Public Health (INSP)	*Food-based dietary guidelines* (year not specified)	NA
Spain	Spanish Agency for Food Safety and Nutrition (AESAN)	*Recomendaciones dietéticas para la población española* (2020) [98]	Evidence-based, food guidance with structured recommendations; vitamin D addressed via nutrient adequacy, not explicit fortification policy

NA—not available (no publicly accessible data).

**Table A2:** Illustrative search terms for national vitamin D fortification policies.

Country	Search Language(s)	Illustrative Search Terms	Official Policy Sources
Austria	German	“Vitamin D”, “Vitamin-D-Anreicherung”, “Anreicherung von Lebensmitteln”, “Vitamin-D-Höchstmenge”, “Verordnung”, “Bundesministerium”	AGES, Federal Ministry of Social Affairs, Health, Care and Consumer Protection, and Austrian legal/regulatory portals
Belgium	Dutch, French, German	“vitamine D”, “vitamine D-fortificatie”, “verrijking van levensmiddelen”; “vitamine D”, “fortification en vitamine D”, “enrichissement des aliments”; “Vitamin-D-Anreicherung”	Federal Public Service Health, Food Chain Safety and Environment, and Belgian legal/regulatory portals
Bulgaria	Bulgarian	“витамин D”, “oбoгатяване с витамин D”, “oбoгатяване на храни”, “наредба”, “министерствo на здравеoпазванетo”	Bulgarian Food Safety Agency and Bulgarian legal/regulatory portals
Croatia	Croatian	“vitamin D”, “obogaćivanje vitaminom D”, “obogaćivanje hrane”, “pravilnik”, “ministarstvo zdravstva”	Ministry of Health of the Republic of Croatia, Croatian Agency for Agriculture and Food (HAPIH), and Croatian legal/regulatory portals
Cyprus	Greek, English	“βιταμίνη D”, “εμπλουτισμός με βιταμίνη D”, “εμπλουτισμός τροφίμων”, “νομοθεσία τροφίμων”; “vitamin D fortification”, “food law”	Public Health Services of the Ministry of Health, State General Laboratory, and Cypriot legal/regulatory portals
Czech Republic	Czech	“vitamin D”, “fortifikace vitamínem D”, “obohacování potravin”, “vyhláška”, “ministerstvo zdravotnictví”	Czech Food Safety Authority (SZÚ), Ministry of Health, and Czech legal/regulatory portals
Denmark	Danish	“D-vitamin”, “berigelse med D-vitamin”, “berigelse af fødevarer”, “bekendtgørelse”, “Fødevarestyrelsen”	Danish Veterinary and Food Administration, Ministry of Food, Agriculture and Fisheries, and Danish legal/regulatory portals
Estonia	Estonian	“D-vitamiin”, “D-vitamiiniga rikastamine”, “toidu rikastamine”, “määrus”, “toiduamet”	Estonian Agriculture and Food Board, Ministry of Regional Affairs and Agriculture, and Estonian legal/regulatory portals
Finland	Finnish, Swedish, English	“D-vitamiini”, “D-vitaminointi”, “elintarvikkeiden täydentäminen”, “asetus”; “berikning med vitamin D”	Ministry of Agriculture and Forestry, Ministry of Trade and Industry, Finnish National Nutrition Council, and Finnish legal/regulatory portals
France	French	“vitamine D”, “fortification en vitamine D”, “enrichissement des aliments”, “arrêté”, “ministère”, “ANSES”	ANSES, DGCCRF, Ministry of Agriculture and Food Sovereignty, and French legal/regulatory portals
Germany	German	“Vitamin D”, “Vitamin-D-Anreicherung”, “Anreicherung von Lebensmitteln”, “Höchstmengen”, “Verordnung”, “BfR”, “BMEL”	German Federal Institute for Risk Assessment (BfR), Federal Ministry of Food and Agriculture (BMEL), and German legal/regulatory portals
Greece	Greek	“βιταμίνη D”, “εμπλουτισμός με βιταμίνη D”, “εμπλουτισμός τροφίμων”, “υπουργείο υγείας”, “νομοθεσία”	Ministry of Health, Hellenic Food Authority (EFET), and Greek legal/regulatory portals
Hungary	Hungarian	“D-vitamin”, “D-vitamin dúsítás”, “élelmiszerek dúsítása”, “rendelet”, “Nébih”	National Food Chain Safety Office (Nébih/NFCSO), Ministry of Agriculture, and Hungarian legal/regulatory portals
Ireland	English	“vitamin D fortification”, “food fortification”, “vitamin D supplementation”, “food law”, “Food Safety Authority of Ireland”	Food Safety Authority of Ireland, Department of Agriculture, Food and the Marine, and Irish legal/regulatory portals
Italy	Italian	“vitamina D”, “fortificazione con vitamina D”, “arricchimento degli alimenti”, “regolamento”, “ministero della salute”	Ministry of Health and Italian legal/regulatory portals
Latvia	Latvian	“D vitamīns”, “bagātināšana ar D vitamīnu”, “pārtikas bagātināšana”, “noteikumi”, “PVD”	Food and Veterinary Service (PVD), Ministry of Agriculture, and Latvian legal/regulatory portals
Lithuania	Lithuanian	“vitaminas D”, “praturtinimas vitaminu D”, “maisto produktų praturtinimas”, “teisės aktas”, “VMVT”	State Food and Veterinary Service (VMVT), Ministry of Agriculture, and Lithuanian legal/regulatory portals
Luxembourg	French, German	“vitamine D”, “fortification en vitamine D”, “enrichissement des aliments”; “Vitamin-D-Anreicherung”, “Lebensmittelanreicherung”	Luxembourg Veterinary and Food Administration (ALVA), ministry/government portals, and Luxembourg legal/regulatory portals
Malta	English, Maltese	“vitamin D fortification”, “food fortification”, “vitamin D supplementation”, “food law”, “public health”	Environmental Health Directorate, Ministry for Health, and Maltese legal/regulatory portals
The Netherlands	Dutch	“vitamine D”, “vitamine D-fortificatie”, “verrijking van levensmiddelen”, “Warenwet”, “Rijksoverheid”	Netherlands Food and Consumer Product Safety Authority (NVWA), Ministry of Health, Welfare and Sport, and Dutch legal/regulatory portals
Poland	Polish	“witamina D”, “fortyfikacja witaminą D”, “wzbogacanie żywności”, “rozporządzenie”, “GIS”, “Ministerstwo Zdrowia”	Chief Sanitary Inspectorate (GIS), Ministry of Health, and Polish legal/regulatory portals
Portugal	Portuguese	“vitamina D”, “fortificação com vitamina D”, “enriquecimento alimentar”, “regulamento”, “Direção-Geral da Saúde”	Directorate-General for Health (DGS), Portuguese Food Safety Authority, and Portuguese legal/regulatory portals
Romania	Romanian	“vitamina D”, “fortificare cu vitamina D”, “îmbogățirea alimentelor”, “ordin”, “ministerul sănătății”	National Sanitary Veterinary and Food Safety Authority (ANSVSA), Ministry of Health, and Romanian legal/regulatory portals
Slovakia	Slovak	“vitamín D”, “fortifikácia vitamínom D”, “obohacovanie potravín”, “vyhláška”, “ministerstvo zdravotníctva”	Ministry of Health of the Slovak Republic, Public Health Authority of the Slovak Republic, and Slovak legal/regulatory portals
Slovenia	Slovenian	“vitamin D”, “obogatitev z vitaminom D”, “obogatitev živil”, “pravilnik”, “ministrstvo za zdravje”	Ministry of Health of the Republic of Slovenia, Administration of the Republic of Slovenia for Food Safety, Veterinary Sector and Plant Protection (AFSVSPP), and Slovenian legal/regulatory portals
Spain	Spanish	“vitamina D”, “fortificación con vitamina D”, “alimentos enriquecidos”, “reglamento”, “ministerio de sanidad”	Spanish Agency for Food Safety and Nutrition (AESAN), Ministry of Health, and Spanish legal/regulatory portals
Sweden	Swedish	“vitamin D”, “berikning med vitamin D”, “berikning av livsmedel”, “föreskrift”, “Livsmedelsverket”	Swedish National Food Agency (Livsmedelsverket), Ministry of Rural Affairs and Infrastructure, and Swedish legal/regulatory portals
United Kingdom	English	“vitamin D fortification”, “fortified foods”, “vitamin D regulation”, “Food Standards Agency”, “UK legislation”	Office for Health Improvement and Disparities (OHID), Food Standards Agency (FSA), and UK legal/regulatory portals
Iceland	Icelandic, English	“D-vítamín”, “auðgun matvæla”, “D-vítamín bætt í matvæli”, “reglugerð”; “vitamin D fortification”	Icelandic Food and Veterinary Authority (MAST), Ministry of Food, Agriculture and Fisheries, and Icelandic legal/regulatory portals
Liechtenstein	German	“Vitamin D”, “Vitamin-D-Anreicherung”, “Lebensmittelanreicherung”, “Verordnung”, “Amt für Lebensmittelkontrolle”	Food Control and Veterinary Office, Liechtenstein government legal-basis pages, and Swiss-linked legal/regulatory sources applicable in Liechtenstein
Norway	Norwegian	“vitamin D”, “beriking med vitamin D”, “beriking av næringsmidler”, “forskrift”, “Mattilsynet”	Norwegian Food Safety Authority (Mattilsynet), Ministry of Health and Care Services, and Norwegian legal/regulatory portals
Switzerland	German, French, Italian	“Vitamin-D-Anreicherung”, “Anreicherung von Lebensmitteln”; “fortification en vitamine D”; “fortificazione con vitamina D”; “AVMO”, “FSVO/BLV”	Federal Food Safety and Veterinary Office (FSVO), Federal Department of Home Affairs (FDHA), and Swiss legal/regulatory portals

Note: Searches combined country names with English and national-language equivalents of terms related to vitamin D, food fortification, legal acts, regulatory limits, and responsible authorities. The terms listed are illustrative and do not represent exhaustive or exact search strings for every country. National-language terms were generated using publicly available translation tools and verified against terminology used on official national authority websites.
